# Deployment of a Fully-Automated Green Fluorescent Protein Imaging System in a High Arctic Autonomous Greenhouse

**DOI:** 10.3390/s130303530

**Published:** 2013-03-13

**Authors:** Talal Abboud, Matthew Bamsey, Anna-Lisa Paul, Thomas Graham, Stephen Braham, Rita Noumeir, Alain Berinstain, Robert Ferl

**Affiliations:** 1 Space Science and Technology, Canadian Space Agency, 6767 route de l'aéroport, Longueuil, QC J3Y8Y9, Canada; E-Mail: alain.berinstain@asc-csa.gc.ca; 2 École de technologie supérieure, 1100 rue Notre-Dame O, Montréal, QC H3C1K3, Canada; E-Mail: rita.noumeir@etsmtl.ca; 3 Horticultural Sciences, University of Florida, Gainesville, FL 32601, USA; E-Mails: matthew.bamsey@ufl.edu (M.B.); alp@ufl.edu (A.-L.P.); robferl@ufl.edu (R.F.); 4 Controlled Environment Systems Research Facility, School of Environmental Sciences, University of Guelph, 50 Stone Road East, Guelph, ON N1G2W1, Canada; E-Mail: tgraham@uoguelph.ca; 5 PolyLAB, Simon Fraser University, 515 W. Hastings Street, Vancouver, BC V6B5K3, Canada; E-Mail: sbraham@sfu.ca; 6 Interdisciplinary Center for Biotechnology Research, University of Florida, Gainesville, FL 32610, USA

**Keywords:** green fluorescent protein, remote sensor, telemetry, plant health, life support, mars, astrobiology, analogue environments, imaging

## Abstract

Higher plants are an integral part of strategies for sustained human presence in space. Space-based greenhouses have the potential to provide closed-loop recycling of oxygen, water and food. Plant monitoring systems with the capacity to remotely observe the condition of crops in real-time within these systems would permit operators to take immediate action to ensure optimum system yield and reliability. One such plant health monitoring technique involves the use of reporter genes driving fluorescent proteins as biological sensors of plant stress. In 2006 an initial prototype green fluorescent protein imager system was deployed at the Arthur Clarke Mars Greenhouse located in the Canadian High Arctic. This prototype demonstrated the advantageous of this biosensor technology and underscored the challenges in collecting and managing telemetric data from exigent environments. We present here the design and deployment of a second prototype imaging system deployed within and connected to the infrastructure of the Arthur Clarke Mars Greenhouse. This is the first imager to run autonomously for one year in the un-crewed greenhouse with command and control conducted through the greenhouse satellite control system. Images were saved locally in high resolution and sent telemetrically in low resolution. Imager hardware is described, including the custom designed LED growth light and fluorescent excitation light boards, filters, data acquisition and control system, and basic sensing and environmental control. Several critical lessons learned related to the hardware of small plant growth payloads are also elaborated.

## 1. Introduction

The use of plants as part of life support systems remains the basis of strategies for sustained human presence in space. Bio-regenerative life support systems have been studied since the early 20th century [1]. The fundamental concept is the use of plants to regenerate air and water while producing food for the crew. The Canadian Space Agency has been involved in assessing the possibility of supporting human presence on the Moon and Mars by deploying greenhouses as plant production system test-beds. There originates the importance of understanding the metabolic issues that can influence plant growth and development in space. Biology is uniquely challenged when exposed to spaceflight and other extraterrestrial environments. These evolutionarily novel environments often require biological organisms to undergo changes in their gene expression to adapt and survive. Like all living organisms, plants continuously perceive their environment and make physiological adjustments based upon that environment. Many adjustments are made at the level of gene expression, thereby enabling an accurate molecular detection system to monitor adjustments to physiology according to environmental needs [2–4]. These adjustments provide the molecular adaptations within the plant cell that allows the plant to survive and even flourish in their new environment. Monitoring the patterns of gene expression to evaluate the response of a plant to a particular environment is an approach used in many studies [5–7]. Coupling these genes with a visible reporter protein permits monitoring of the environment in real-time [8,9]. The reporter protein Green Fluorescent Protein (GFP) is very well suited to be utilized as an environmental monitor [10,11]. In the orbital and planetary surface environments, where the return of samples is extremely difficult, GFP monitoring can be accomplished via telemetry. In addition, the imaging process does not affect plant growth and development, nor does it compromise plant health [11,12].

The Transgenic Arabidopsis Gene Expression System (TAGES) was developed as a set of biosensor plants engineered with reporter gene constructs developed for evaluating the biological response of Arabidopsis to spaceflight and other novel environments [11–13]. This is an example of the direct use of an organism to determine the quality of its environment. Many commercial systems are available for imaging and capturing fluorescence, however none are available for data capture and transmission in space-related systems. Near term biological experiments in space are likely to be autonomous or robotic missions that would require information to be transmitted to Earth by telemetry [14]. In addition, compromises among internal atmospheric pressure, structure, associated infrastructure and crew-time, will likely require autonomous telemetric monitoring.

Experience from a second generation TAGES imaging system (TIS-II) and the first deployed within the Arthur Clarke Mars Greenhouse (ACMG) [14], led to the redesign and deployment of the imaging system described here (TIS-III). The TIS-II deployed in 2006, had plant growth lights that were positioned behind the Petri dish. Due to the considerable amount of heat emitted from these LEDs, condensation was formed on the Petri dish that subsequently obstructed the imaging area. The TIS-II had a power and control box that was separate from the nominal greenhouse power controllers. In addition, several of the imager components required different voltages as well AC current input, which conflicted with the greenhouse renewable energy system. Furthermore, only manual controls were available to adjust growth / excitation light intensity and power the system on or off. A computer connected to the imager controlled and commanded the system which made it complicated and power intensive. TIS-II captured a maximum of 250, non-compressed, 1,280 × 1,280 pixel GFP images. Due to these described issues it was not possible to leave the imager to run autonomously and to send commands and data telemetrically via satellite link after the field season.

This article describes the design and development of a second generation prototype fluorescent imager (TIS-III) deployed in the ACMG at the Mars Institute's Haughton Mars Project Research Station on Devon Island (Nunavut) in the Canadian High Arctic [15–17]. The project was initiated to take a demonstrated laboratory imaging system and advance the necessary software, hardware and communication system to permit the use of this technology in an extreme, operational environment. The study is intended not as a biological test of the environment, but as a test of the imaging hardware in that environment. In comparison to the described TIS-II imager, TIS-III included modified and repositioned grow lights to improve plant growth and limit condensation. The GFP LED board was redesigned; the control box and camera were replaced by a National Instruments Compact FieldPoint controller and camera that could be connected and controlled via the greenhouse software and communication systems. The imager used a 24 VDC connected to the readily available and compatible greenhouse renewable energy system.

## 2. Materials and Methods

Arabidopsis (*Arabidopsis thaliana*) is a model organism studied in biology laboratories and has strong spaceflight heritage. Arabidopsis has many advantages for genomic studies; the genome has been fully sequenced, the plants are small in stature with a rapid life cycle (about 6 weeks from seed to seed) and produce abundant progeny. There is also a wealth of mutant lines and Arabidopsis can be easily transformed through the use of *Agrobacterium tumefaciens* [18]. These benefits make it the best understood candidate with respect to the reactions and adaptation to environmental stresses, and when transformed with reporter genes, Arabidopsis has proven a useful tool for the development and deployment of biological sensors to evaluate patterns of gene expression in response to remote and novel environments [11,13,19].

The green fluorescent protein (GFP), used as a tag, is a protein that emits bright green fluorescence when exposed to blue light. The term GFP stems from the protein first isolated from jellyfish [10]. The TAGES reference GFP, entitled S65T, has a primary excitation peak at 488 nm. Its emission peak is at 512 nm, which is in the lower green wavelength portion of the visible spectrum [9,11]. The main advantage of GFP is that the excitation, imaging and labeling procedure does not affect plant growth and development, while other methods require the addition of a substrate and often sacrifice the plant [14]. The GFP reporter used in imager calibration was CaMV35s::GFP. The 35s promoter from the Cauliflower Mosaic Virus (CaMV) is constitutively expressed in plants and provides a brightly fluorescing positive control. The 35S promoter is not influenced by the environments presented in this study, and this GFP construct was used strictly as a fluorescent marker to evaluate the hardware and fluorescent data collection. For the purposes of this test, the CaMV35s::GFP construct was inserted into the WS Arabidopsis ecotype [11]. The purpose of the present experiments is strictly an analysis of the imaging hardware and its capabilities. Therefore only this one GFP reporter was used to characterize the imager and no data are presented that seek to evaluate the physiological response of the plants.

### 2.1. Fundamental Excitation System Requirements

A wide range of incident radiation wavelengths (300 < λ < 500 nm) will result in energy transfer and can drive GFP excitation resulting in emission of green fluorescent light; however, the greatest quantitative fluorescence response occurs when the incident excitation λ = 488 nm. Although 488 nm light has the biggest quantitative effect of fluorescence transfer, wavelengths below this peak will also be absorbed. This knowledge leads to the selection of excitation sources, taking into account all wavelengths that produce light at or below 488 nm and keeping the power efficiency in the equation of the excitation source. The excitation is greatest at 488 nm but also very close to the short end of the emission spectrum of the green GFP at 502 nm. This means that to capture the emission of GFP the excitation source should be totally blocked from entering the imaging system. The excitation system should not emit light with wavelengths higher than 493 nm. This can be accomplished either by selecting lights that will not illuminate, or by filtering all wavelengths, above 493 nm.

### 2.2. Fundamental Emission Image Capture Requirements

GFP emission occurs within the green portion of the visible spectrum with peak emission at approximately 512 nm. However, the fluorescence emitted by S65T covers a considerable bandwidth, meaning that valuable information is collected not only at 511 nm, but at a wide range surrounding this peak. The system should have an image sensor capable of capturing wavelengths of 502 nm and longer. Wavelengths longer than 502 nm allow the use of yellow and red fluorescent proteins, and also the potential to capture natural chlorophyll fluorescence. It is important to point out that there is a strict constraint that the wavelengths produced by the excitation system do not overlap with the emission of fluorescence captured by the imaging system. Contamination of the emission light by the excitation source can result in considerable loss of information with significant negative implications for the resulting science output.

### 2.3. Arthur Clarke Mars Greenhouse

The Arthur Clarke Mars Greenhouse (ACMG) is a biological life support systems test facility developed to study and understand automated greenhouse production in extreme environments. Established in 2002, the ACMG became part of the Haughton-Mars Project (HMP), which is an international field research project that participates in numerous disparate studies. Located on Devon Island close to the Haughton crater in the Canadian High Arctic, HMP uses the remote polar desert and uninhabited island as a terrestrial environmental analogue for Mars and Moon. The site's geologic topography and biological aspects promote a distinctive research and operations environment suitable to the elaboration of knowledge, technologies, and field based operational methodologies that could be a significant step to successful long duration human space missions. The ACMG does not have all the functionality of a replicated closed loop life support system to be deployed on Mars or the Moon. On the other hand, it supports extreme environment related scientific and operational research, providing an improved understanding of how remote and semi-autonomous plant production systems could one day be operated. The ACMG is composed of a DC power system, plant growth system, environmental control system, local network, communication system, data acquisition and control system [15,16]. The greenhouse operates autonomously throughout the year, with the researchers only on-site during the month of July when the crops are harvested and reseeded [20]. The ACMG was the test bed for the TIS-II GFP imager deployed in 2006 and the TIS-III GFP imager in 2010.

The ACMG power comes from a DC renewable energy system composed of six solar panels of 110 W [peak] each and can provide a total of 660 W during a sunny day in the summer. In addition, the greenhouse is powered with two wind turbines, each with a peak output of 400 W. The solar panels and wind turbines are connected to a battery bank through a set of charge controllers. The greenhouse power consumption varies between 150 W, during the spring/fall season, and 10 to 25 W when the greenhouse is in idle mode. All greenhouse sensors, relays, Ethernet cameras and the TIS-III imager are controlled and synchronized by a programmable logic controller with an intelligent Ethernet control interface. The information collected by the controllers, sensors and cameras is accessed by an independent embedded Linux command and control computer and sent via satellite to an autonomous mission operations computer server system at Simon Fraser University (SFU). Upon reception of the data, the information is processed and stored, and then made available to the researchers through the Internet [16]. The autonomous mission operations server at SFU can determine failure modes from received data and provide basic autonomous commanding via satellite to the embedded computer in the ACMG, which can further command the controllers, and/or power distribution system. Also, the operations center can send commands to the data acquisition and control system via the SFU mission operations server to modify the mode of operation of the greenhouse and the TIS-III imager.

## 3. GFP Imager

The TAGES Imaging System-III (TIS-III) is a modified and upgraded version of TIS-II, a second generation fluorescent imaging payload that was designed to collect GFP expression data in real-time, during a spaceflight experiment [12,14]. The main objective of TIS-III was to design an imaging device to conduct remote, semi-autonomous experiments, within the extreme environment of a High Canadian Arctic planetary analogue site. The TIS-III was developed as an autonomous, remotely operated and controlled plant GFP imaging system connected to the communications infrastructure of the ACMG to manage dataflow from the imaging device. The upgrades were necessary to resolve several issues: (1) The need to reduce energy consumption was required as the imager was to be deployed in a remote area with a limited energy source. The excitation lights required 4 A of current and needed 10.5 VDC so each image capture sequence demanded 42 W. In addition, laboratory tests showed that the grow lights where consuming over 3 A at their max potential of 24 V, which is over 72 W; (2) The earlier TIS-II imager power supply was large, heavy and in some instances unreliable as many of the components utilized differing voltages and those voltages were not directly available through the nominal ACMG power system; (3) The camera could not be controlled remotely and without a computer connected to it. In addition, only one image resolution/compression could be set for capturing. The power supply, grow lights and excitation lights were all controlled with mechanical switches or knobs and required human presence to make any changes; (4) The grow light board behind the sample tray generated too much heat, and thus was having a negative influence on plant growth and the imaging target was often obstructed due to condensation forming on the front end of the sample tray. To solve these constraints with the TIS-II, many parts had to be designed and built new.

### 3.1. Hardware

**Figure 1.**
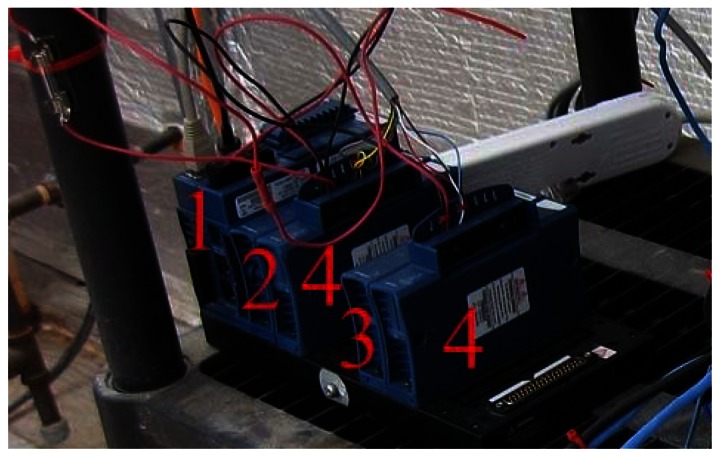
The controller setup as deployed within the ACMG on Devon Island. Shown are cFP-2120 (**1**); cFP-PWM-520 (**2**); cFP-RLY-421 (**3**) and cFP-CB-1 connector blocks (**4**).

**Figure 2.**
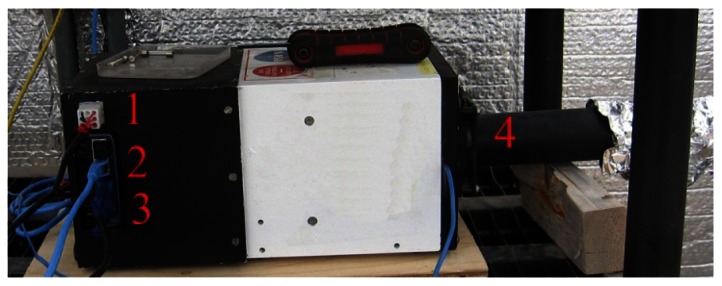
Side view of the TIS-III as installed. Grow lights and fan inputs (**1**); camera Ethernet connection (**2**); camera power cable (**3**); cooling pipe (**4**).

**Figure 3.**
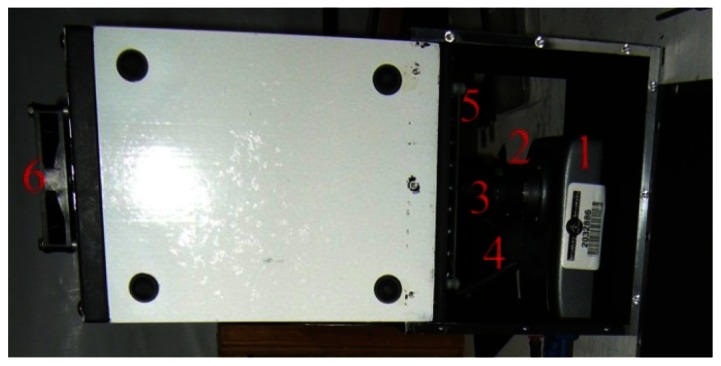
Top view of the installed TIS-III NI-1744 Camera (**1**); Computer M1214-MP lens (**2**); camera guide bracket (**3**); camera Light control connector (**4**); excitation light board (**5**); cooling fan (**6**).

The greenhouse has a local network that links different components through an Ethernet hub. The control of systems, namely the plant growth system, the environment control system, the power system, the communication system, and the data acquisition system, are performed by National Instruments (NI) Field Point controllers (cFP-2120) through Ethernet ports and coded with LabVIEW. To ensure compatibility, autonomy and simple integration to the ACMG, all TIS-III components were chosen and designed to be powered with 24 VDC. In addition, a cFP-2120 controller (Figure 1), LabVIEW programmed, is used to control and collect data from the imager. For the same reasons, the NI-1744 smart camera was selected as the capturing device. It has spot on features needed, that is, could be powered with a 24 VDC power supply, monochrome 1,280 × 1,024 CCD 8 bit pixel depth image sensor, built-in lighting controller, Ethernet ports and programmable with LabVIEW Real-Time Module or with Vision Builder AI. NI extension modules cFP-PWM-520 and cFP-RLY-421 (Figure 1) served as intensity and relay controllers for the grow lights, camera and cooling fan (Figures 2 and 3). A 1.44 W (24 VDC × 0.06 A) cooling fan was positioned behind the Petri dish to keep the plants cool and avoid image obstructions due to condensation on the front face of sample Petri plates. To further reduce the possibility of condensation on this front face, a pipe bringing cooler air from the exterior of the greenhouse to the integrated TIS-III fan was also deployed (Figure 2). The power budget of every component that constitutes the TIS-III is displayed in Table 1. Since not all components are active at the same time, power consumption depends on the state of the imager. Typically, the grow lights are on for eight hours per day and with them total power consumption equals 17.62 W (6.1 W + 10.08 W + 1.44 W). During an image capture sequence, the camera is powered on for approximately five minutes, the excitation LEDs for two minutes and two of the three grow light boards for one minute (sequence is set every six hours).

### 3.2. Software

Communication with other parts of greenhouse is via Ethernet and IP protocols. The Linux-based embedded computer communicates with the controllers and cameras and uses an SDX-1100 modem that communicates with an MSAT satellite to transmit information south to the autonomous mission operations server at SFU, while commands are sent north.

**Figure 4.**
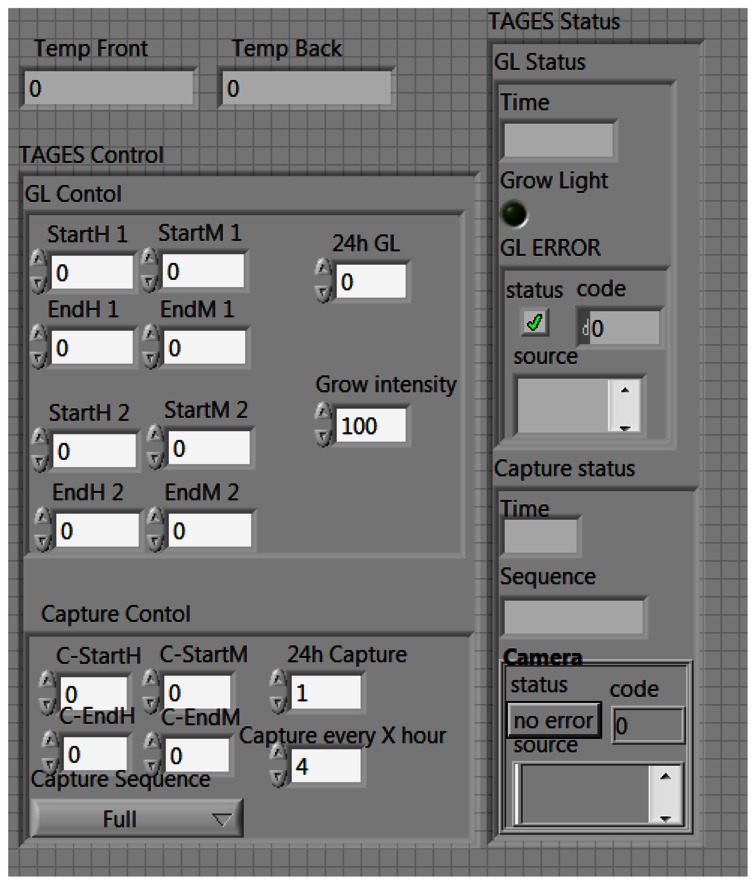
LabVIEW TIS-III control panel.

**Figure 5.**
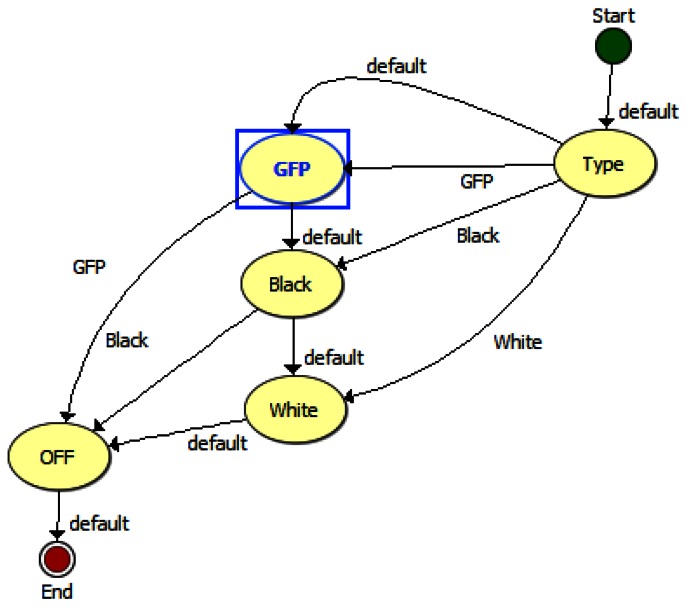
VBAI Inspection State Diagram (TIS-III capture sequence logic).

The TIS-III program was directly integrated in the ACMG software, both coded with LabVIEW, which made the imager part of the greenhouse by receiving commands and sending information via the same communication system. Figure 4 presents the TIS-III software control panel: grow light intensity is controlled by setting the PWM value (0 to 100%), the light scheduling can be set to start and end twice any time during the day or stay on for 24 h. The time and frequency of the image capture sequence can also be set and sent by the user via satellite link. For compatibility with the greenhouse software, all inputs are integer values. The capture sequences and excitation lighting control are controlled by the NI-1744 smart camera and coded with Vision Builder for Automated Inspection (VBAI). Figure 5 shows the VBAI Inspection State Diagram. The controller and camera communicate via shared variables through the network. When the Capture Sequence variable is set to Full, as in Figure 4, the VBAI State Diagram will follow the default path (Figure 5) and the camera will capture three types of images:

(1) GFP: The grow lights are off and excitation lights are on. This image captures only the fluorescence emitted from the sample.
(2) Black: All lights are off. This image is subtracted from the GFP image to remove any imperfections the CCD (e.g., bad pixels).
(3) White: The two grow light boards are on. This image produces a regular “white light” image.

All images files are saved in two formats. The Tagged Image File Format (TIFF) [21] is a flexible format that normally saves 8 bits or 16 bits per color (8 bits equal 256 level of gray in our case) and uses a lossless compression. For the implemented camera the file size of 1.25 MB per image when the resolution is set to 1,280 × 1,024. The second format is Joint Photographic Experts Group (JPEG) [22], a “lossy” compression method that compresses data by discarding (losing) some of it. The process aims to minimize the amount of data that needs to be sent via satellite. The camera stores the data on the cFP-2120 controller's 4 GB compact flash card and named using the following format; ddmmyy-HHMMSS-type-Image.tif (or jpg). Due to the low speed of the communication link between the greenhouse and the satellite, only the JPEG images are transmitted over the satellite link for an initial review and to confirm plant status. The TIFF images are collected the next year field season for a full science study.

### 3.3. Grow Lights

To redesign the grow light board, different red and blue LEDs were selected based on recent studies [23–26] and the two types of red and blue LEDs were chosen based on the peak absorption wavelengths for chlorophyll ***a*** measured around 430 nm and 660 nm and for chlorophyll ***b*** around 454 nm and 643 nm. Since green light is primarily reflected by plants, only a small number of green LEDs were incorporated into the grow light board so as to permit more nominal ‘white light’ imaging and to complete the photosynthesis spectra. Laboratory light distribution tests were conducted to find a pattern that was considered to best illuminate the plant with all the needed light. Table 2 presents the LEDs selected and their specified wavelength outputs.

**Figure 6.**
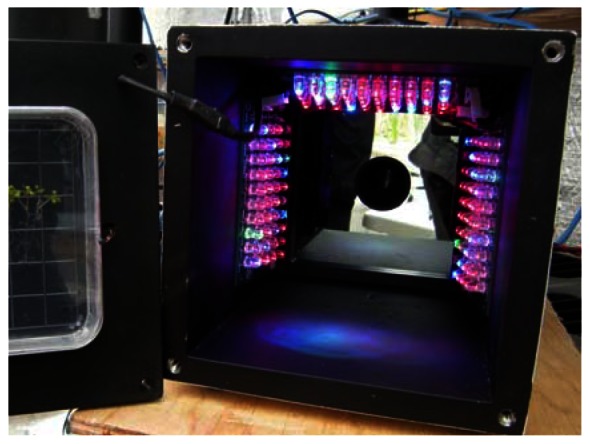
Grow lights boards and positioning within the TIS-III.

To not obstruct the camera and the excitation lights, three small grow light boards were placed inside the TIS-III, in front of the Petri dish, two on the sides and one on the top. The lights rays focussed on the plants by lifting one end of each board by one cm, as shown in Figure 6.

**Figure 7.**
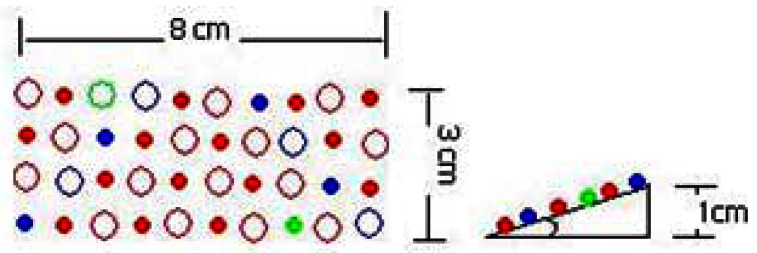
Grow light distribution pattern, size and installation angle.

To minimize the complexity of the grow light printed circuit boards (PCB), LEDs of each row were connected in series and designed to use a 24 VDC power source, a voltage source readily available in the ACMG. The current in each row is limited by the LED that has the lowest maximum current. A ballast resistor for each row was added to limit the current to the maximum allowed in that row. The full red LED maximum current is 40 mA and all the others are above that. From Ohm's law the appropriate resistance was calculated to be R = 0.7 V/40 mA = 17.5 Ohms or higher. In addition, a potentiometer was added in case a higher voltage is needed. Each grow light board draws 140 mA, and thus the three consume approximate 10.08 W (420 mA × 24 V) at full intensity. Table 3 presents the voltage and distribution of LEDs of a single grow light board. The color pattern was distributed in such a way to cover all the plant area with the entire range of required wavelengths and angle was added to maximize the incident light on plants as shown in Figure 7.

Being able to control the grow light intensity is a necessity; too much light will result in increased heat dissipation leading to heat stress responses in plants; too little light and the plants won't grow properly. Two types of dimming are available: analog and by PWM. With analog dimming, X% brightness is achieved by applying X% of the maximum current to the LED. The main downside to this process is LED color shifting, in which the emitted wavelengths are different depending on the applied current. This shift results in a failure to achieve the objective of emitting the desired wavelength. PWM dimming is accomplished by applying full current to the LED at a reduced duty cycle. For X% brightness, full current is applied at an X% duty cycle. The frequency of the PWM signal must be above 100 Hz to ensure that the PWM pulsing is not visible to the human eye. The TIS-III grow light intensity is controlled by PWM using an NI extension module cFP-PWM-520 module. Each grow light board is connected to a channel that can supply up to 1 A.

### 3.4. Fluorescent Excitation Lights

**Figure 8.**
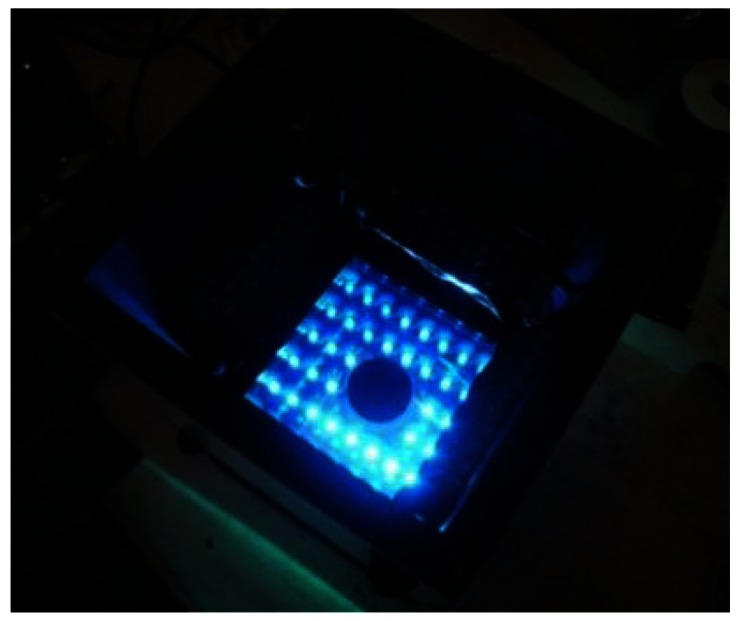
TIS-III excitation LEDs behind a band-pass filter.

The excitation light board was also designed to use a 24 VDC power supply even though it's powered and controlled using the NI-1744 smart camera current source output. LEDs are current-driven devices whose illumination is proportional to their current. Their current can be controlled in two ways. The first method, which was used to control the grow light board, is to use the LED V-I curve to determine what voltage needs to be applied to the LED to generate the desired current. This is usually achieved by applying a voltage source and using a ballast resistor. However, this method has a major drawback especially for the excitation source. Any change in LED forward voltage generates a change in LED current. The need to be consistent is essential since the board is designed to excite the green fluorescent proteins and subsequently capture their fluorescence. Changes in the LED current will produce changes in the LED intensity, therefore variations in the captured fluorescence intensity. The preferred method of regulating LED current is to drive the LED with a constant-current source which is readily available on the NI-1744 smart camera. The current source is constant and eliminates changes in current due to variations in voltage, which translates into a constant LED brightness. The excitation board has eight parallel rows of six LEDs in series. The Roithner LED (B56L5111P) sustains a voltage of 3.8 V at a current of 50 mA. The camera current source is set to a maximum of 400 mA (8 rows × 50 mA) which dissipate a total of 9.12 W during an excitation sequence. Figure 8 shows the powered excitation light board, behind a narrow band-pass filter to avoid any overlap and contamination of the emitted fluorescence, which would contaminate the resulting science. The TIS-II salvaged filter has a spectrum spread of 470 nm to 488 nm, with a band-pass mid-point of 475 nm. In addition, another recovered long pass filter (over 500 nm) was modified to fit in the TIS-III camera above the CCD and behind the optical lens.

## 4. Results and Discussion

**Figure 9.**
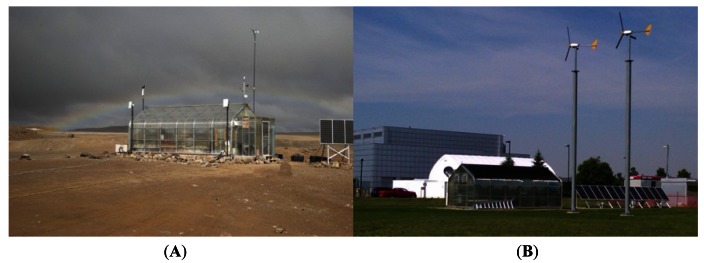
ACMG (**A**) and the CSA development greenhouse (**B**).

All TIS-III hardware and software went through a month of verification and validation prior to the deployment to the Arctic greenhouse (Figure 9(A)). The output of the excitation lights (filtered light) and grow lights was verified using a USB4000 spectrometer, CC-3 cosine corrector, one meter QP400-1-UV-VIS optical fiber and SpectraSuite software from Ocean Optics. Measurements were taken using a calibrated FLUKE multimeter to verify the excitation lights current source output. An oscilloscope (Tektronix TDS3054B) was used to verify the PWM grow light voltage frequency, ratios and voltage output. After calibrating the lens to focus on the biological sensor and adjusting the camera settings to capture the maximum amount of fluorescence (without saturation), tests were conducted to verify that only the emission lights were captured. The described validation process was implemented to confirm that the developed imaging system met all the initial design requirements. The process confirmed that all the components where communicating and performing nominally. In addition, to minimize technology deployment risk, maximize confidence in any new modifications and to improve researchers work efficiency, every new or improved system was validated at a replica of the ACMG greenhouse at the Canadian Space Agency (CSA) headquarters in Longueuil, Quebec (Figure 9(B)) [14]. A simulated deployment was performed for the TIS-III at the CSA greenhouse and confirmed compatibility.

**Figure 10.**
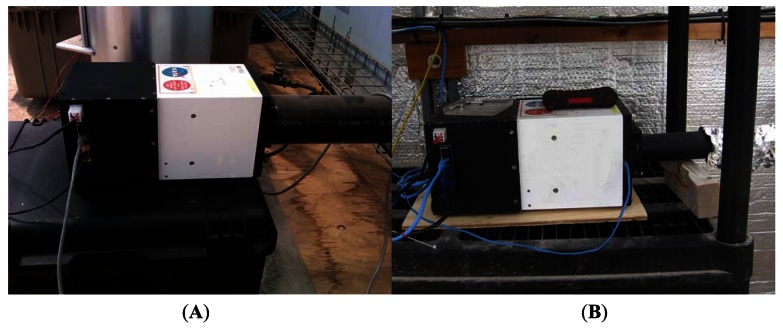
CSA validation phase within the CSA development greenhouse (**A**); nominal operations within the ACMG (**B**).

During installation, it was realized that, unlike the CSA development greenhouse, the ACMG did not have a properly regulated 24 VDC output. The 24 VDC was connected directly to the DC renewable energy system battery bank. By examining the data collected by the greenhouse sensors of the previous year, it indicated that the 24 VDC unregulated line varies between ∼23.5 V to peaks of ∼33 V. The imager hardware was examined to study the possibility of modifying parts not capable of withstanding the high voltage peaks. The cFP-2120 controller data sheet indicated that the power supply range was 11–30 VDC and since similar controllers were used from the beginning of the greenhouse deployment in 2002 [16], it was concluded that it was safe to use the unregulated 24 VDC line on the TIS-III controller. The NI-1744 camera data sheet specified that the smart camera accepted power within the range of the industry standard IEC 1311 input power specification (24 V + 20% – 15% with an additional allowance for an AC peak of + 5%), at 33 V (short and infrequent peaks), it's slightly above that specified by the manufacturer. In addition, the camera was powered for only a few minutes every few hours. With this information it was also decided that it was safe to connect the camera directly to the unregulated line. Since the excitation board was connected to the camera current source (direct drive), not only the lights were safe, but their intensity would not fluctuate in the event of power variation as the camera would adjust to apply the same current set by the user. The only element that had to be further developed due to the issue of unregulated power was the grow light board and this was accomplished by increasing the ballast resistor values that limit the current. It was mentioned previously that a resistor for each row was added to limit the current to the maximum allowed in that row. Using this information, the specifications of the LEDs that limited the current in each row were consulted to determine the new ballast resistor values. It was also considered that since the voltage peaks are in bursts of short periods and as LED specifications indicated that they would withstand a higher current in short duty cycles, the new ballast resistor values were calculated using the higher current limits. This solution decreased the maximum grow light intensity, but the intensity was still maintained within the range of the Arabidopsis requirements. After the changes were implemented, the imager was connected to the greenhouse Ethernet hub, powered by the 24 V unregulated line and became part of the greenhouse sensor suite. Instructions and information were controlled by the greenhouse computer system. Images were stored on-site in the TIS-III controller and only JPEG files were sent via satellite with the rest of the ACMG data stream. Figure 10 shows TIS-III during the test phase conducted at the CSA (A) development greenhouse and during actual ACMG deployment (B).

**Figure 11.**
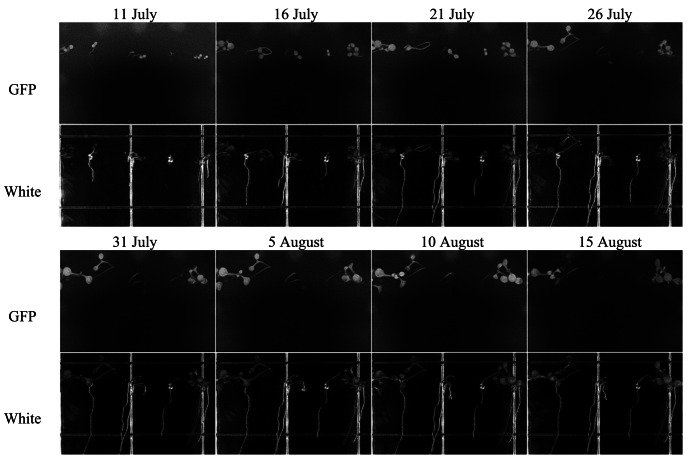
Automated 2010 fall operations run showing captured TIS-III and remotely downloaded plants expressing GFP and regular white light images. Images were cropped and processed to adjust contrast and brightness for publishing and printing purposes. Images were captured every 6 hours and the presented images reflect 35 days of growth with an interval of five days.

TIS-III was deployed in the ACMG in July 2010 to help understand the operational constraints and to experiment with the integration of a fully automated imaging sensor within the ACMG system for a full year without any on-site human intervention. After deployment of the imager and departure of the field crew, TIS-III was set to capture a set of images (GFP, Black, and White) every six hours and the grow lights were operated at 100% intensity for 24 h a day. From 10 July to 24 September low resolution images were collected and transferred via the greenhouse satellite connection to Simon Fraser University. During this period, the greenhouse operated in its nominal fall operations mode where only the fall crop trays were watered (spring crop trays were inactive) [16]. Figure 11 shows a set of images taken during the fall run from 11 July to 15 August, when the plants are in full growth and with a chosen interval of five days between images to better visualize the demonstrated growth.

**Figure 12.**
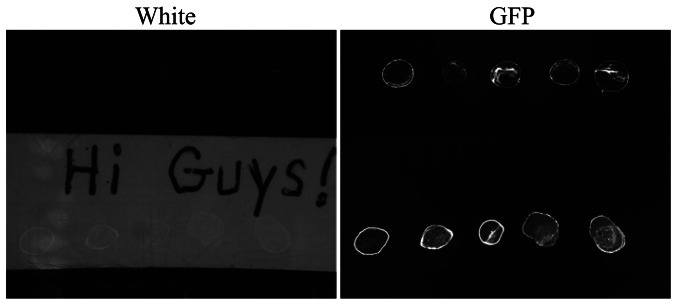
Spring season repair confirmation images downloaded south via satellite link.

**Figure 13.**
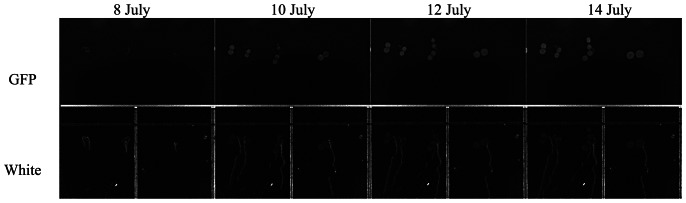
2011 Automated spring run showing captured and remotely downloaded plants expressing GFP and regular white light images. Images were cropped and processed to adjust contrast and brightness for publishing and printing purposes. Images were captured every six hours and the presented images reflect approximately six days of growth with an interval of two days.

Following the successful operation of the imager during fall 2010, ACMG commands were sent on September 24, via satellite link, to put the greenhouse into dormant mode, which allows the system to survive over the dark and cold season [15]. All systems, including TIS-III, were shutdown to conserve power, with the exception of the communication system to conserve energy. On 1 May 2011, greenhouse power was restored and TIS-III was reactivated. Still, with no human presence, TIS-III resumed collecting data and images and this information was sent south. It was then discovered that something was not operating nominally, in particular while the GFP images were fine, the regular white images were black. Following an extensive troubleshooting effort conducted over the communications link it was concluded that a remote recovery was not likely. The research team determined that a small pre-field season visit would be conducted to repair the imager while at the same time replacing the biological sample contained within. This would permit a spring operations period to be conducted with the imager during the nominal ACMG spring operations phase. The imager recovery visit permitted the scientific principle investigators of the imager, who have themselves had substantial Shuttle and ISS payload experience, the opportunity to act in a contrasting role and in this instance, implement the overall research team's developed crew and imager repair procedures (cf. the investigators aiding in directing astronauts to conduct their developed on-orbit science/hardware operations). The repair deployment was conducted by a team of two and the conducted over a one day period (early July). The deployment team carried with them procedures for troubleshooting a suite of potential failure modes, tools and hardware for these repairs and a satellite phone to communicate with the ACMG operations team in the south. During this visit the crew traced the imager failure to a blown PWM module and in tandem to its replacement, installed a 24 VDC regulator on the line feeding the imager. Confirmation of the repair (Figure 12) was soon obtained by the operations team when imaging data was successfully downloaded over the satellite link and nominal operations subsequently recommenced as shown in Figure 13.

## 5. Conclusions

An imaging device that captures *in situ* biological signals and translates these signals into measures of plant stress would be a valuable diagnostic tool for bio-regenerative life support systems. Numerous systems are available for capturing fluorescence in the laboratory but are restricted to the collection of data locally. However, biological experiments in space or in remote regions will likely be autonomous and include robotic operations where telemetric commands and data collection is a necessity because of crew unattainability and safety. The development of the fully integrated imaging system that captures fluorescence images is a significant step that stretches the technical capability of sending diagnostics in a telemetric fashion from an extra-terrestrial location. TIS-III was developed with new features including the custom designed LED grow and excitation light boards, filters, data acquisition, control system and basic environmental control, all powered with a single power source readily available at the northern greenhouse. In addition, TIS-III software was coded and integrated to the ACMG operating system, which made imager hardware and software fully compatible with the ACMG systems and contributed to the successful imager deployment as part of the sensor suite in the ACMG. Also contributing to the successful deployment was the test and simulated operations of the imager within the CSA development greenhouse. The usefulness of such an integrated test, one that included both validation of hardware as well as validation of science output is one that other such remote/extreme environment hardware deployments should consider.

TIS-III was the first imager that ran autonomously in the un-crewed greenhouse, its deployment in the ACMG and its subsequent full year of operations have demonstrated the feasibility of plant diagnostic systems that transmit and receive data by satellite link allowing near-real time monitoring and control of space biology experiments and bio-regenerative life support systems.

## Figures and Tables

**Table 1.** TIS-III power budget.

**NI-1744 Camera**	**cFP-2120 Controller**	**PWM-520 Module**	**RLY-421 Module**	**Grow Lights**	**Excitation Lights**	**Cooling Fan**
6.48 W	6.1 W	∼ 0 W	∼ 0 W	10.08 W	9.12 W	1.44 W

**Table 2.** Selected Roithner Lasertechnik LEDs utilized in the developed grow light boards and their respective wavelength ranges.

**Color**	**Item #**	**Range (nm)**	**Peak (nm)**
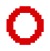	ELD-670-524	660–680	670
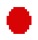	ELD-650-523	640–660	650
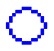	LED450-06	430–450	445
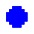	5B4HCA-H	460–470	465
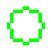	B5-433-B505	490–510	500
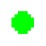	5G4HCA-H	520–530	525

**Table 3.** LED distribution and voltages.

	**Voltage of Row 1**	**Voltage of Row 2**	**Voltage of Row 3**	**Voltage of Row 4**
LED 1	3.2	2.3	1.9	2.3
LED 2	1.9	3.3	2.3	1.9
LED 3	2.3	1.9	3.2	3.0
LED 4	1.9	2.3	1.9	3.3
LED 5	2.3	1.9	2.3	1.9
LED 6	1.9	2.3	1.9	2.3
LED 7	2.3	1.9	2.3	3.2
LED 8	3.0	2.3	3.3	1.9
LED 9	2.3	3.2	1.9	2.3
LED 10	3.3	1.9	2.3	1.9
**V Total**	**24.4**	**23.3**	**23.3**	**24**

## References

[1] Wheeler R.M. (2010). Plants for human life support in space: From Myers to Mars. Gravit. Space Biol..

[2] Ahuja I., De Vos R.C., Bones A.M., Hall R.D. (2010). Plant molecular stress responses face climate change. Trends Plant Sci..

[3] Cushman J.C., Bohnert H.J. (2000). Genomic approaches to plant stress tolerance. Curr. Opin. Plant Biol..

[4] Sultan S.E. (2010). Plant developmental responses to the environment: Eco-Devo insights. Curr. Opin. Plant Biol..

[5] Chen W., Provart N.J., Glazebrook J., Katagiri F., Chang H.S., Eulgem T., Mauch F., Luan S., Zou G., Whitham S.A. (2002). Expression profile matrix of Arabidopsis transcription factor genes suggests their putative functions in response to environmental stresses. Plant Cell.

[6] Kant P., Gordon M., Kant S., Zolla G., Davydov O., Heimer Y.M., Chalifa-Caspi V., Shaked R., Barak S. (2008). Functional-genomics-based identification of genes that regulate Arabidopsis responses to multiple abiotic stresses. Plant Cell Environ..

[7] Kilian J., Whitehead D., Horak J., Wanke D., Weinl S., Batistic O., D'Angelo C., Bornberg-Bauer E., Kudla J., Harter K. (2007). The AtGenExpress global stress expression data set: Protocols, evaluation and model data analysis of UV-B light, drought and cold stress responses. Plant J..

[8] Plautz J.D., Day R.N., Dailey G.M., Welsh S.B., Hall J.C., Halpain S., Kay S.A. (1996). Green fluorescent protein and its derivatives as versatile markers for gene expression in living Drosophila melanogaster, plant and mammalian cells. Gene.

[9] Stewart C.N. (2001). The utility of green fluorescent protein in transgenic plants. Plant Cell Rep..

[10] Sheen J., Hwang S., Niwa Y., Kobayashi H., Galbraith D.W. (1995). Green-fluorescent protein as a new vital marker in plant cells. Plant J..

[11] Manak M.S., Paul A.-L., Sehnke P.C., Ferl R.J. (2002). Remote sensing of gene expression in Planta: Transgenic Plants as monitors of exogenous stress perception in extraterrestrial environments. Life Support Biosph Sci..

[12] Paul A.-L., Murdoch T., Ferl E., Levine H.G., Ferl R. (2003). The TAGES imaging system: Optimizing a green fluorescent protein imaging system for plants. SAE Tech. Paper.

[13] Paul A.-L., Schuerger A.C., Popp M.P., Richards J.T., Manak M.S., Ferl R.J. (2004). Hypobaric biology: Arabidopsis gene expression at low atmospheric pressure. Plant Physiol..

[14] Paul A.-L., Bamsey M., Berinstain A., Braham S., Neron P., Murdoch T., Thomas G., Ferl J.R. (2008). Deployment of a prototype plant GFP imager at the Arthur Clarke Mars Greenhouse of the Haughton Mars Project. Sensors.

[15] Bamsey M., Berinstain A., Graham T., Neron P., Giroux R., Braham S., Ferl R., Paul A.-L., Dixon M. (2009). Developing strategies for automated remote plant production systems: Environmental control and monitoring of the Arthur Clarke Mars Greenhouse in the Canadian High Arctic. Adv. Space Res..

[16] Giroux R., Berinstain A., Braham S., Graham T., Bamsey M., Boyd K., Silver M., Lussier-Desbiens A., Lee P., Boucher M. (2006). Greenhouses in extreme environments: The Arthur Clarke Mars Greenhouse design and operation overview. Adv. Space Res..

[17] Lee P., Braham S., Boucher M., Schutt W.J., Briggs G., Glass B., Gross A., Hine B., McKay C.P., Hoffman S.J. Haughton-Mars Project: 10 Years of Science Operations and Exploration Systems Development at a Moon/Mars Analog Site on Devon Island, High Arctic.

[18] Koornneef M., Meinke D. (2010). The development of Arabidopsis as a model plant. Plant J..

[19] Paul A.-L., Daugherty C.J., Bihn E.A., Chapman D.K., Norwood K.L.L., Ferl R.J. (2001). Transgene expression patterns indicate that spaceflight affects stress signal perception and transduction in arabidopsis. Plant Physiol..

[20] Bamsey M., Graham T., Stasiak M., Berinstain A., Scott A., Vuk T.R., Dixon M. (2009). Canadian advanced life support capacities and future directions. Adv. Space Res..

[21] Tagged Image File Format. http://en.wikipedia.org/wiki/Tagged_Image_File_Format#cite_ref-0.

[22] JPEG. http://en.wikipedia.org/wiki/JPEG.

[23] Goins G.D., Yorio N.C., Sanwo M.M., Brown C.S. (1997). Photomorphogenesis, photosynthesis, and seed yield of wheat plants grown under red light-emitting diodes (LEDs) with and without supplemental blue lighting. J. Exp. Botany.

[24] Porra R.J., Thompson W.A., Kriedemann P.E. (1989). Determination of accurate extinction coefficients and simultaneous equations for assaying chlorophylls a and b extracted with four different solvents: Verification of the concentration of chlorophyll standards by atomic absorption spectroscopy. BBA Bioenerg..

[25] Schurr U., Walter A., Rascher U. (2006). Functional dynamics of plant growth and photosynthesis-from steady-state to dynamics-from homogeneity to heterogeneity. Plant Cell Environ..

[26] West-Eberhard M.J., Smith J.A., Winter K. (2011). Photosynthesis, reorganized. Science.

